# Les démoniaques dans l'art: Charcot and the “hysterical saints”

**DOI:** 10.1055/s-0042-1759709

**Published:** 2022-12-28

**Authors:** Léo Coutinho, Marlon Wycliff Caeira, Luciano de Paola, Olivier Walusinski, Francisco Eduardo Costa Cardoso, Plinio Marcos Garcia de Lima, Hélio A. Ghizoni Teive

**Affiliations:** 1 Universidade Federal do Paraná, Serviço de Neurologia, Unidade de Distúrbios do Movimento, Curitiba PR, Brazil.; 2 Universidade Federal do Paraná, Programa de Pós-Graduação em Medicina Interna, Grupo de Doenças Neurológicas, Curitiba PR, Brazil.; 3 Universidade Federal do Paraná, Serviço de Neurologia, Unidade de Epilepsia, Curitiba PR, Brazil.; 4 Private Practive, Brou, France.; 5 Universidade Federal de Minas Gerais, Hospital das Clínicas, Serviço de Neurologia, Departamento de Medicina Interna, Belo Horizonte MG, Brazil.; 6 Universidade Federal do Paraná, Serviço de Neurologia, Curitiba PR, Brazil.

**Keywords:** Hysteria, History of Medicine, Religion and Medicine, Art, Epilepsy, Histeria, História da Medicina, Religião e Medicina, Arte, Epilepsia

## Abstract

Professor Jean-Martin Charcot was the founder of clinical neurology and one of the prominent researchers in the field of hysteria in the 19 ^th^ century. His book *Les démoniaques dans l'art* is a representation of hysterical symptoms in religion and religious art. This paper aims to discuss Charcot's descriptions of hysteria in religion and his “hysterical saints”.

## Resumo

Professor Jean-Martin Charcot foi o fundador da neurologia clínica e um dos pesquisadores mais proeminentes no campo da histeria durante o século XIX. Seu livro *Les démoniaques dans l'art* é uma representação dos sintomas histéricos na religião e arte religiosa. Esse artigo objetiva discutir as descrições de Charcot de histeria na religião e seus “santos histéricos”.

## INTRODUCTION

The term hysteria is derived from the Greek word *Hysterikós* , meaning “relative to the womb”. This correlation was established in ancient Greece because most cases of hysteria occurred in women, but only in the 16 ^th^ century the term hysteria was regularly applied to designate such functional disorders. 1 2 3 4

In the middle age, hysteria gained a religious connotation, and the hysterical phenomenology was attributed to demonic possession or witchcraft. A similar situation to the one experienced by patients with other neurological conditions, such as stroke and epilepsy. 1 2 3 4

**Figure. 1:**
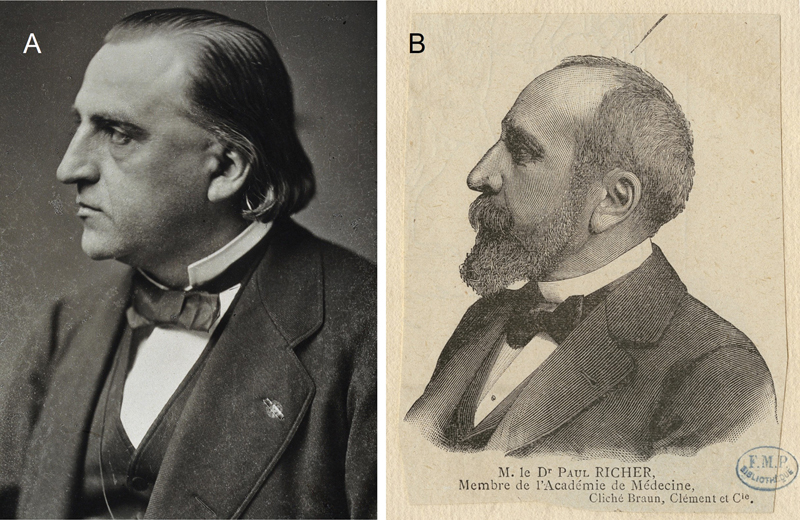
(A) Professor Jean-Martin Charcot (1825–1893). (B) Paul Richer (1849–1933). Source A: The Wellcome collection. Licensed under a public domain mark. Source B: Bibliothèques d'Université Paris Cité. Licensed under a public domain mark.

In the 19 ^th^ century, Jean-Martin Charcot (1825–1893) ( Figure. 1A ) developed an interest in the subject of hysteria, becoming one of the major researchers in the field. Aside from its pathological roots, Charcot was also interested in the historical and cultural aspects of hysteria, particularly its relationship to religion and religious art. 5 6

This interest motivated Charcot to publish a book in 1887, alongside his pupil Paul Richer (1849–1933) ( Figure. 1B ): *Les démoniaques dans l'art* . In this work, illustrated by Richer, they discussed how hysteria was represented in religious art, presenting works of art featuring some of the saints of the catholic church that were possibly presenting a hysterical event. 7 This paper discusses *Les démoniaques dans l'art* , emphasizing Charcot's “hysterical saints.”

## MOTIVATIONS AND INFLUENCE FOR *LES DÉMONIAQUES DANS L'ART*

It is curious that Charcot took the burden of untangling the cloudy subject of hysteria since he had no prior interest in mental illnesses. His contact with the works of Pierre Janet and Briquet, besides the influence of Desirée Bourneville, one of the few of his pupils with experience with alienism, were determinants for his interest in hysteria. 8 9 10

Charcot had strong political and religious standpoints. An anti-clerical and fierce defendant of laicism in all scientific investigations, Charcot opposed religious intervention in scientifical affairs. 5

Charcot was very fond of art, with a predilection for the classics. A gifted artist himself, he produced numerous self-portraits, drawings, and sketches. 5 In 1874, Charcot participated as chair of the thesis *Permanent deformations of the hand from the point of view of medical semiotics* , by Henri Meillet with drawings of Richer. Impressed by the quality of his art, Charcot invited Richer to join his service at the *Salpêtrière* . Richer's artistical prowess also contributed to his own thesis, *Études cliniques sur l'hystéro-épilepsie ou grande histérie* . 11

*Les démoniaques dans l'art* was preceded by the start of Bourneville's work, the *Bibliothèque diabolique* , nine books, published between 1882 and 1902. 10 In the third volume of his collection, Bourneville published writings attributed to Johan Wier (1515–1588), the first to consider the victims as sick and to oppose the use of exorcism. 12 Bourneville's initiative is possibly the first step of Charcot-Richer's work.

Another worthy contribution was Edward Jorden's publication *A brief discourse of a disease called the suffocation of the mother* , in 1603. This book is considered a turning point in the understanding of hysteria, presenting it as a disease instead of a religious event. 13

## THE GRAND HYSTERICAL ATTACK AND THE “HYSTERICAL SAINTS”

In *Les démoniaques dans l'art* , Charcot presents his classical description of the grand hysterical attack, divided in four periods (Epileptoid, Clownism, *Attitudes passionnelles* and Final delirium). 7 Paul Regnard and Bourneville had previously documented the attack with photography, publishing with clinical observations in the *Iconographie photographique de La Salpêtrière* . 14

Charcot describes a variant of the third period of the grand hysterical attack, with a predominance of the feeling and constant ecstatic facial expression. The patient is typically quiet, presenting a delusional speech, and might present negative sensorial symptoms, such as achromatopsia, blindness, and anesthesia. These sensorial symptoms are referred to as *stigma* . Hallucinations are common, often with a religious nature. This phenomenon is commonly described as an epiphanic, religious event, sometimes possessing an erotic connotation. 7

**Figure. 2:**
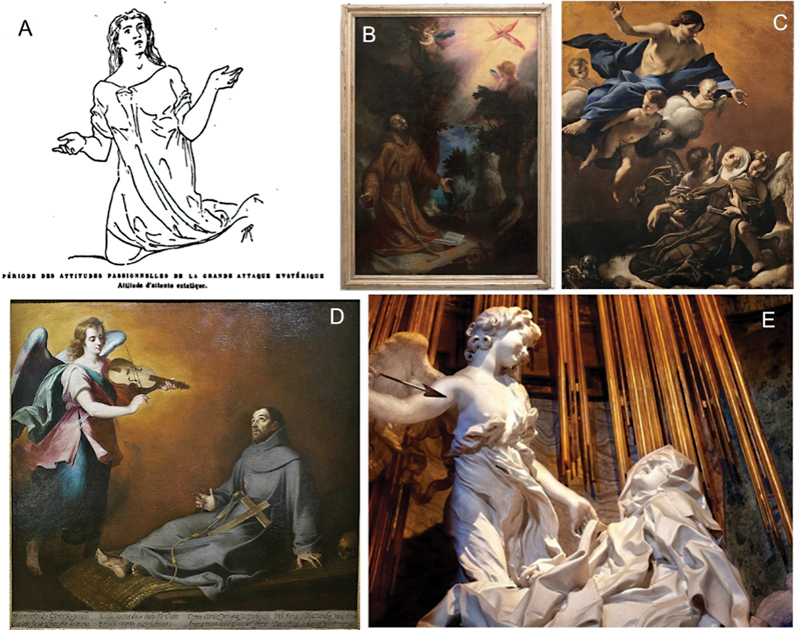
(A) *Sainte Catherine de Sienne en extase* . (B) Saint Francis receives the stigmata, by Cigoli. (C) Ecstasy of Saint Margaret of Cortona, by Giovanni Lanfranco. (D) The ecstasy of Saint Francis of Assisi, by Bartolomé Esteban Murillo. (E) The ecstasy of Saint Teresa, by Gian Lorenzo Bernini. Source A: *Les démoniaques dans l'art* . 7 Licensed under a public domain mark. Source B: Le gallerie degli Uffizi. Licensed under a public domain mark. Source C: Web Gallery of art. Licensed under a public domain mark. Source D: Real Academia de Bellas Artes de San Fernando. Licensed under a public domain mark. Source E: From Prof. Francisco Cardoso's private archives.

Charcot illustrates the ecstatic crisis with the case of *Sainte Catherine de Sienne* , providing a copied fragment of the fresco that decorates Saint-Dominique Church, in Sienna ( Figure. 2A ). The picture portrays *Sainte Catherine* in an attitude of ecstatic contemplation, with a facial expression of joy. Other representations of the “Hysterical saints” are mentioned by Charcot, such as “ *Saint François recevant les stigmates* ” ( Figure. 2B ) “ *Sainte Marguerite de Cordoue en extase* ” ( Figure. 2C ), and *“Saint François en extase* ” ( Figure. 2D ), but the respective images were not provided by Charcot and Richer. 7

A lacking honorable mention is Gian Lorenzo Bernini's sculpture “The Ecstasy of Saint Teresa” ( Figure. 2E ), the prototypical representation of an ecstatic posture. Years later, Bourneville published a book by Hippolyte Rouby (1860–1920), *L'hystérie de Sainte-Thérèse* , in his *Bibliothèque diabolique* , reinforcing the relevance of Bernini's work in depicting ecstatic phenomenology. 15 The sensations related to this ecstatic event were documented by the saint, who described the appearance of an angel, bearing a golden spear with a flaming point, piercing Teresa's heart several times, raising a sensation whose “sweetness […] is so extreme that one cannot possibly wish it to cease” 16 .

This sexual and orgasmic description is commonplace among the ecstatic religious events, as seen in the illustrations of Saint Catherine and Saint Francis. Charcot pointed this out, defining this uniformity as almost scientific, praising such artistic rigor. 7

In modern times, these ecstatic events were also related to epileptic activity, typically secondary to non-dominant temporal lobe abnormalities, sometimes with a sexual and orgasmic phenomenology, giving rise to the term “Orgasmic epilepsy” 17 .

Charcot and Richer also present examples of religious art illustrating the other periods of the grand hysterical attack, such as the limb circumduction movements of the epileptoid period in a fragment of Deodat Delmont's *La transfiguration* , the opisthotonos of the clownism period in an image of Jesus performing an exorcism from *La Bible de Picart* , and the bizarre dystonic postures of the final delirium in a tableau from Saint-Ambroise church, at Genoa. 7

In conclusion, the depiction of religious events as a possible manifestation of functional disorders was unusual to the conservative 19 ^th^ -century Parisian society, despite prior contributions in the literature. Charcot's publication of *Les démoniaques dans l'art* is a tribute to laicism in science, demonstrating his unswerving respect for neurology.
